# Editorial: Development of circadian clock functions, volume II

**DOI:** 10.3389/fnins.2024.1453328

**Published:** 2024-07-16

**Authors:** Jihwan Myung, Rae Silver, Jeff R. Jones, Takahiro J. Nakamura, Daisuke Ono

**Affiliations:** ^1^Graduate Institute of Mind, Brain, and Consciousness, Taipei Medical University, Taipei, Taiwan; ^2^Department of Psychology, Columbia University, New York, NY, United States; ^3^Department of Neuroscience and Behavior, Barnard College of Columbia University, New York, NY, United States; ^4^Department of Biology, Texas A&M University, College Station, TX, United States; ^5^Laboratory of Animal Physiology, School of Agriculture, Meiji University, Kawasaki, Japan; ^6^Stress Recognition and Response, Research Institute of Environmental Medicine, Nagoya University, Nagoya, Japan; ^7^Department of Neural Regulation, Nagoya University Graduate School of Medicine, Nagoya, Japan

**Keywords:** circadian clock, development, aging, entrainment, synchronization, suprachiasmatic nucleus (SCN)

In multicellular organisms like ourselves, there exists not only a single circadian clock but rather a network of clocks throughout the body that operate in coordination. The central pacemaker of this system is the suprachiasmatic nucleus (SCN). The SCN is itself synchronized to the environment by local external light-dark cycles and it then communicates this information to the rest of the body. However, this is not a steady state relationship: As our bodies undergo changes throughout their developmental trajectory, there are corresponding adjustments in circadian organization. These adjustments necessitate consideration not only of external synchronizing cues, but also internal circadian cues that entrain and organize clocks throughout the body.

Because the proper maintenance of circadian rhythms is important in early life, addressing the developing circadian clock is critical in the neonatal intensive care unit (NICU). This is particularly pertinent for preterm infants prior to gestational week 30, who have not yet opened their eyes, making photic entrainment unreliable (Arimitsu et al.). Consequently, there is a need to consider alternative methods of entrainment, such as internal entraining signals: It is not known however, which signaling molecules mediate the internal synchronization of circadian clocks. Glucocorticoids are one potential candidate that can be especially relevant during fetal development, as their receptor response can be gated by time (Lehmann et al.). Another potential candidate are Opn4 (melanopsin) aptamers, or melapts. Given that the SCN receives light input directly from melanopsin-expressing intrinsically photosensitive retinal ganglion cells (ipRGCs), melapts can potentially intervene in light-stimulated phase shifts of circadian clocks (Nakazawa et al.). These candidate signaling molecules could have therapeutic implications for circadian entrainment in artificial environments such as the NICU.

The development of circadian clock systems appears to undergo stages of unification (from absence to presence of synchronization) and specification (organized phases through maturation) (Myung et al., 2021). Carmona-Alcocer et al. work tracing the trajectories of nascent SCN neuronal development through the patterns of neuropeptide expression reveals the delayed specification of arginine vasopressin (AVP) and vasoactive intestinal peptide (VIP) neurons. Surprisingly, this time course of specification exhibits some sexual dimorphism. In addition, the synchronization among clock neurons follows a developmental trend that deteriorates with old age. This loss of synchronization may be due to changes in the neurotransmitter GABA, one of the primary interneuronal signaling molecules in the SCN, during aging. Olde Engberink et al. find that GABA, canonically an “inhibitory” neurotransmitter, becomes less inhibitory and progressively more excitatory with age, which effectively affects the coupling strength among neurons within the SCN clock network. In aged mice, this results in changes to the spectral power of circadian rhythmicity and a longer period in the SCN oscillation. The mechanism behind this has a simple theoretical explanation: synchronization is driven by the frequencies of component oscillators, not their periods. If their period distribution of individual oscillators within the SCN is symmetric, the frequency distribution becomes skewed, making the synchronized frequency differ from the mean frequency (Myung et al.).

The molecular clock itself—the transcriptional-translational feedback loop intrinsic to single cells—also changes during development. Indeed, the core clock component *Cryptochrome* (*Cry*) has previously been found to have developmental criticality (Ono et al., 2013). The *Cry1* isoform specifically influences circadian period in behavior across development, summarized as: “when *Cry1* expression was higher for longer [in early development], the free-running period [of locomotor activity] was longer” (Schirmer et al.).

Finally, the development of the circadian clock has effects on physiology in both model organisms and importantly, in humans. A human's intrinsic circadian period is reflected in their chronotype (morningness-eveningness). In a two-sample Mendelian randomization analysis, Yang et al. find a causal relationship between a morning chronotype in humans and the structural features of the cerebral cortex. Intriguingly, in mice, electroencephalography (EEG) recordings of cortex reflect these structural changes and show age-dependent circadian changes with circadian power weakened with age (Masuda et al.). Developing circadian clocks also have significant relevance to neurovascular function (Mitchell and Gillette).

In conclusion, circadian clocks are present in all developing systems and they appear with different strengths and specifications, enabling coordination of multiple subsystems in the brain and the body. The precise mechanisms that determine how such coordination is achieved through various interactions will continue to provoke ongoing investigation.

## Author contributions

JM: Writing – original draft, Writing – review & editing. RS: Writing – review & editing. JJ: Writing – review & editing. TN: Writing – review & editing. DO: Writing – review & editing.
